# Predicting AURKA as a novel therapeutic target for NPC: A comprehensive analysis based on bioinformatics and validation

**DOI:** 10.3389/fgene.2022.926546

**Published:** 2022-08-22

**Authors:** Chaobin Huang, Lin Chen, Yiping Zhang, Liyan Wang, Wei Zheng, Fengying Peng, Yuanji Xu

**Affiliations:** ^1^ Department of Radiation Oncology, Clinical Oncology School of Fujian Medical University, Fujian Cancer Hospital, Fuzhou, Fujian, China; ^2^ College of Clinical Medicine for Oncology, Clinical Oncology School of Fujian Medical University, Fujian Cancer Hospital, Fuzhou, Fujian, China; ^3^ Department of Pathology, Clinical Oncology School of Fujian Medical University, Fujian Cancer Hospital, Fuzhou, Fujian, China

**Keywords:** *AURKA*, nasopharyngeal carcinoma, therapeutic targets, cell cycle, immune infiltration

## Abstract

This study comprehensively explored the clinical function of Aurora kinase A (*AURKA*) gene in nasopharyngeal carcinoma (NPC) and analyzed its potential as a therapeutic target in cancer. Data were downloaded from GEO, STRING, GTEx, and CellMiner databases, and subjected to multiple bioinformatic analyses, including differential expression analysis, WCGNA, gene ontology (GO), Kyoto encyclopedia of genes and genomes (KEGG), gene set enrichment analysis (GSEA), gene set variation analysis (GSVA), miRNA-hub gene regulatory network analysis, immune cell infiltration, and drug sensitivity analysis. In-depth analysis of *AURKA* gene expression in NPC and its corresponding clinicopathological features was performed to explore its potential as a therapeutic target. Moreover, *AURKA* gene expression in NPC was validated by qRT-PCR in 21 NPC tissues and 17 normal nasopharyngeal epithelial tissues. *AURKA* was highly expressed in NPC tissues. Enrichment analysis of *AURKA* and its co-expressed hub genes indicated their oncogenic role in NPC and their potential involvement in cancer-promoting processes through histone kinase activity and microtubule motility activity, cell cycle, and p53 signaling pathways. *AURKA* high expression group had greater infiltration of neutrophils, macrophages M2, and dendritic cells resting and less infiltration of T cells CD4^+^ naïve and T cells γδ. Drug susceptibility analysis found that dacarbazine, R-306465, vorinostat, and other antitumor drugs that act on the cell cycle were closely related to *AURKA*. qRT-PCR verified the high expression of *AURKA* in NPC tissues (*p* < 0.05). We confirmed upregulation of *AURKA* in NPC tissues. Our results support an oncogenic role of *AURKA* in the context of NPC, and indicate its potential role as a novel therapeutic target.

## Introduction

Nasopharyngeal carcinoma (NPC) is the most common head and neck malignancy originating from the nasopharyngeal epithelium, predominantly in the fossa of Rosenmüller (Kamran et al., 2015; Chua et al., 2016; Yosra et al., 2019). Globally, newly-diagnosed NPC accounted for 0.7% of all cancer diagnoses in the year 2020, with 133,354 cases (Sung et al., 2021). Radiation therapy is the first-choice treatment for early NPC. However, post-radiotherapy 5-years survival rate of patients with advanced NPC is only 50%, owing to local recurrence and distant metastasis (Kong et al., 2010). Despite some progress in the research on the therapeutic targets for NPC, the improvement in 5-years survival rate is less than satisfactory (Wu et al., 2015). Further research on the pathogenesis of NPC and the identification of key causative genes are key imperatives to unravel novel therapeutic targets and improve the prognosis of NPC patients.

A series of cell function experiments and *in vivo* studies in mouse models by Zhao et al. revealed that circTMTC1 promotes NPC deterioration through the miR-495-MET-eIF4G1 axis (Zhao et al., 2022). Zhu et al. found a close association between *ALDH1B1* expression and prognosis of locally-advanced NPC (Zhu et al., 2022). In addition, studies have suggested that *p38γ* is a key oncogene and an important therapeutic target for NPC (Yin et al., 2022), and that Circ_0028007 promotes NPC progression by adsorbing miR-656-3p and increasing *ELF2* expression (Ma and Li, 2022). These findings indicate that targeted therapy for NPC is a promising area for further research. However, there is a need for in-depth characterization of the specific mechanisms of these findings.

Aurora kinase A (*AURKA*) (also referred to as *AIK, STK7*, and *PPP1R47*) is a protein-coding gene that is widely expressed in multiple tissues. High expression of *AURKA* has been reported in various tumors (Yan et al., 2016; Wu et al., 2018; Wang-Bishop et al., 2019; Yi et al., 2021). Most notably, studies have explored the biological function of *AURKA* in human cancers, such as diffuse large B-cell lymphoma, glioblastoma, gastric, cervical, colorectal, and liver cancers (Shen et al., 2019; Liu et al., 2021; Mesquita et al., 2021; Nguyen et al., 2021; Wang and Sun, 2021; Wu et al., 2021). In addition, inhibition of *AURKA* was shown to result in a significant reduction of *GPX4* and induction of ferroptosis in tumor cells (Gomaa et al., 2019). Therefore, we chose *AURKA* as the research object and explored the potential functions and mechanisms of *AURKA* through various analyses.

Despite AURKA has been shown to be an oncogene in many cancers, till date, the role of *AURKA* in NPC is not well characterized. At present, the targeted therapy of nasopharyngeal carcinoma has entered a bottleneck period, and overcoming it has become a clinical focus and difficulty. Therefore, in-depth study of the potential mechanism of AURKA in NPC is crucial, which may open up a new direction for targeted therapy of NPC. This study provides important support for exploring the clinical function of *AURKA* in NPC and its potential as a new therapeutic target.

## Materials and methods

### Sample collection and preprocessing

Thirty-eight tissue samples, including NPC (*n* = 21) and normal nasopharyngeal epithelium (*n* = 17) were obtained from patients at the Fujian Cancer Hospital between June 2019 and December 2020. None of the study participants had received any treatment prior to nasopharyngoscopy. Written informed consent was obtained from all subjects. The Biomedical Ethics Committee approved this study at the Fujian Cancer Hospital (No. SQ 2019-068-01). The study procedures complied with the principles of the Declaration of Helsinki. The patient’s pathological staging and typing was according to the American Joint Committee on Cancer (AJCC) classification criteria.

### Date download and processing

The NPC expression profiling datasets GSE12452 (Dodd et al., 2006; Sengupta et al., 2006; Hsu et al., 2012) and GSE13597 (Bose et al., 2009)were downloaded from the GEO (https://www.ncbi.nlm.nih.gov/geo/) database using the GEOquery package of R software (version 4.0.3, http://r-project.org/) (Meltzer and Davis, 2007). The GSE12452 dataset includes data of 31 tumor samples and 10 normal samples, and the GSE13597 dataset includes data of 25 tumor samples and 3 normal samples. The limma package was used to perform data normalization on the GSE12452 and GSE13597 datasets (Ritchie et al., 2015).

### Screening of differentially expressed genes

Tumor and normal samples were screened for DEGs in the GSE12452 and GSE13597 datasets using the limma package (*p* < 0.05, |log2FC|>1). The intersection of these DEGs was obtained. The *AURKA* gene was selected as the research object. The expression of *AURKA* in GSE12452 and GSE13597 was plotted using the ggpubr (https://CRAN.R-project.org/package=ggpubr) package, and the discrimination efficiency of *AURKA* in the two datasets was analyzed using the ROCR package (Sing et al., 2005). Finally, the expression of *AURKA* in human tissues was detected in the GTEx database.

### Analysis of *AURKA* correlation

We divided tumor samples into high- and low-expression groups based on median *AURKA* expression, and the limma package was used to screen the DEGs (*p* < 0.05, |log2FC|>1). The 3D principal component analysis (PCA) plots were drawn using the scatterplot3d package (Mächler and Ligges, 2003) to show the similarity between the two sets of samples. The heatmap and ggplot2 packages were used to draw heat map and volcano map, respectively, to show the overall expression and differential expression of *AURKA*-related genes.

### WCGNA

We first performed a co-expression network analysis of genes using the R package WGCNA (Langfelder and Horvath, 2008) and then performed network construction and module identification by topological overlap metric (TOM). Finally, the genes in the most significant modules were intersected with the previously analyzed DEGs to identify the hub genes.

### Functional enrichment analysis

We performed Gene Ontology (GO) and Kyoto Encyclopedia of Genes and Genomes (KEGG) enrichment analysis on hub genes using the cluster profile package (Yu et al., 2012). In addition, gene set enrichment analysis (GSEA) and gene set variation analysis (GSVA) were performed on the gene expression matrix using the clusterProfiler package and the GSVA package (Hnzelmann et al., 2013), respectively.

### PPI interaction network, miRNA-hub gene regulatory network analysis

The STRING protein-protein interaction database was used to analyze the interaction relationship of the hub genes. After the software exported the results, the core genes were further screened using the CytoHubba plugin in Cytoscape (Chin et al., 2014). In addition, Hub gene-miRNA regulation analysis was performed by Networkanalyst (http://www.networkanalyst.ca/NetworkAnalyst), and miRNA-target gene prediction was performed based on the minimum number of network connections. The miRNA-hub gene regulatory network was drawn by Cytoscape software.

### Immune infiltration and correlation analysis

The proportions of 21 immune cells in the dataset samples were predicted by CIBERSORT (http://CIBERSORT.stanford.edu/) (Newman et al., 2015) and the LM22 eigengene matrix. We assessed the abundance of 21 immune cells in the GSE12452 dataset through the CIBERSORT package. The samples were divided into high and low expression groups based on the median *AURKA* expression. Finally, by integrating *AURKA* expression information, the correlation between *AURKA* and immune infiltrating cells was assessed using Pearson correlation coefficient (*p* < 0.05).

### Drug sensitivity analysis

The mRNA expression profile and drug activity data of the *AURKA* gene were downloaded from the CellMiner database (https://discover.nci.nih.gov/cellminer/). The correlation between *AURKA* and compound sensitivity was assessed by Pearson correlation analysis (*p* < 0.05).

### qRT-PCR validation of the expression of *AURKA*

*AURKA* expression in 21 NPC and 17 normal tissues was verified through qRT-PCR. The clinical data for qRT-PCR of patients with NPC in our institution was shown in Supplementary Table S1. The primers of *AURKA* are listed in Supplementary Table S2 and were synthesized by BioSune (Shanghai, China). The internal reference gene was 18S-rRNA. We used RTIII All-in-One Mix and dsDNase (Monad Biotech Co., Ltd., Shanghai, China) to reverse-transcribe 1 µg of total RNA into cDNA. The qRT-PCR validation was performed on the StepOnePlus Real-Time PCR System (Applied Biosystems, Thermo Fisher Scientific Co., Ltd., US) using Hieff^®^ qPCR SYBR^®^ Green Master Mix, High Rox (Yeasen, Biotechnology Co. Ltd., Shanghai, China). The reaction system was: 95 °C for 10 min, then 41 cycles of 95°C for 15 s and 60°C for 1 min, last 95°C for 15 s.

## Results

### Data preprocessing and gene screening

**FIGURE 1:**
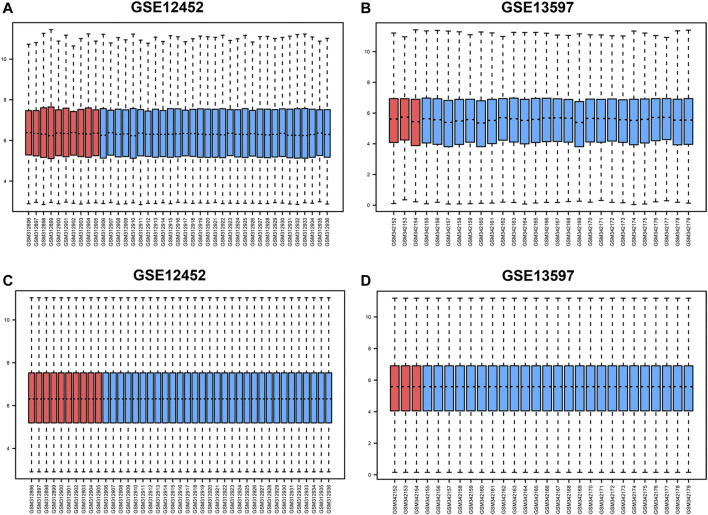
Box diagram before and after standardization of GSE12452 and GSE13597 datasets. **(A–B)** Box diagrams before standardization of GSE12452 and GSE13597; **(C–D)**. Box diagrams after standardization of GSE12452 and GSE13597. Blue represents tumor samples, and red represents normal samples. NPC, nasopharyngeal carcinoma; GEO, Gene Expression Omnibus.

The gene expression matrix for the GSE12452 and GSE13597 datasets before and after normalization are presented as box plots (Figures 1A–D). After preprocessing the data, we used the limma package to perform differential analysis on GSE12452 and GSE13597 gene expression data. Finally, 731 DEGs were identified in the GSE12452 dataset, and 1,096 DEGs were identified in the GSE13597 dataset. After screening, *AURKA* was found to be differentially expressed in both datasets.

### *AURKA* expression detection and functional analysis

**FIGURE 2:**
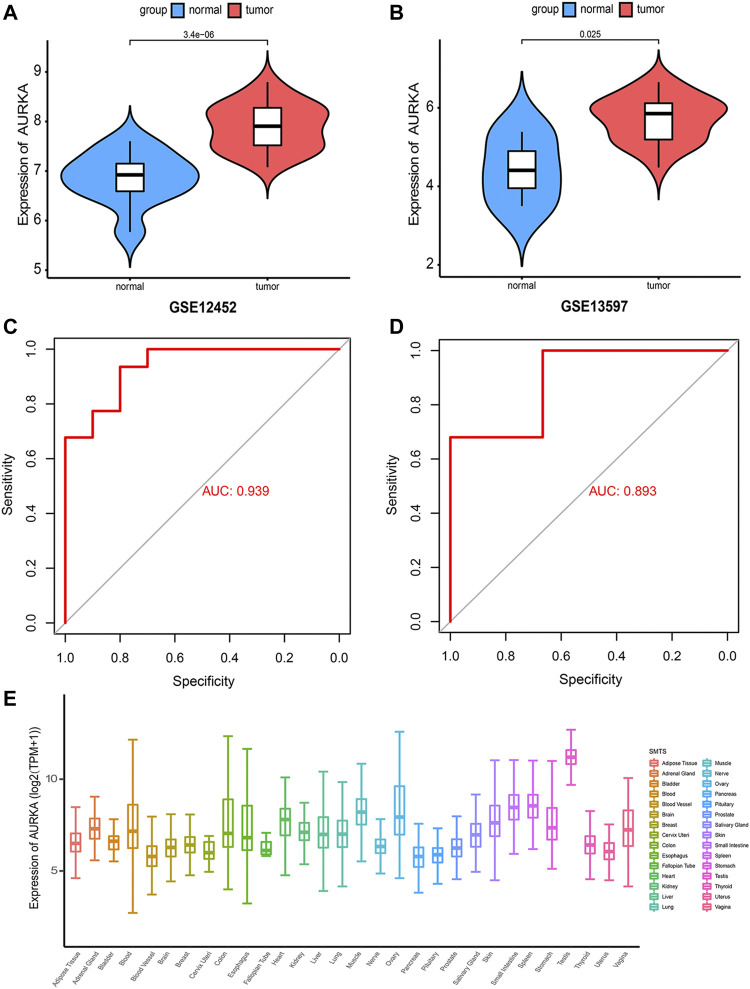
Expression detection of *AURKA* gene **(A,B)**. Violin plot showing the expression of *AURKA* gene in GSE12452 and GSE13597 datasets; **(C,D)**. ROC curve showing that the two groups have a high degree of discrimination, with an AUC of 0.939 in the GSE12452 group and 0.893 in the GSE13597 group; **(E)**. Expression of *AURKA* in human tissues.

After selecting *AURKA* as the target gene, we compared the expression of *AURKA* between tumor samples and normal samples in the GSE12452 and GSE13597 datasets. The results showed significantly greater expression of *AURKA* in tumor tissues compared with normal tissues (*p* < 0.05) (Figures 2A,B). On receiver operating characteristic (ROC) curve analysis, the AUC of the GSE12452 group and GSE13597 group was 0.939 and 0.893, respectively. This showed that the two groups were well-differentiated (Figures 2C,D). Subsequently, we detected the expression of *AURKA* in human tissues through the GTEx database and found that its expression was highest in the testis (Figure 2E).

**FIGURE 3:**
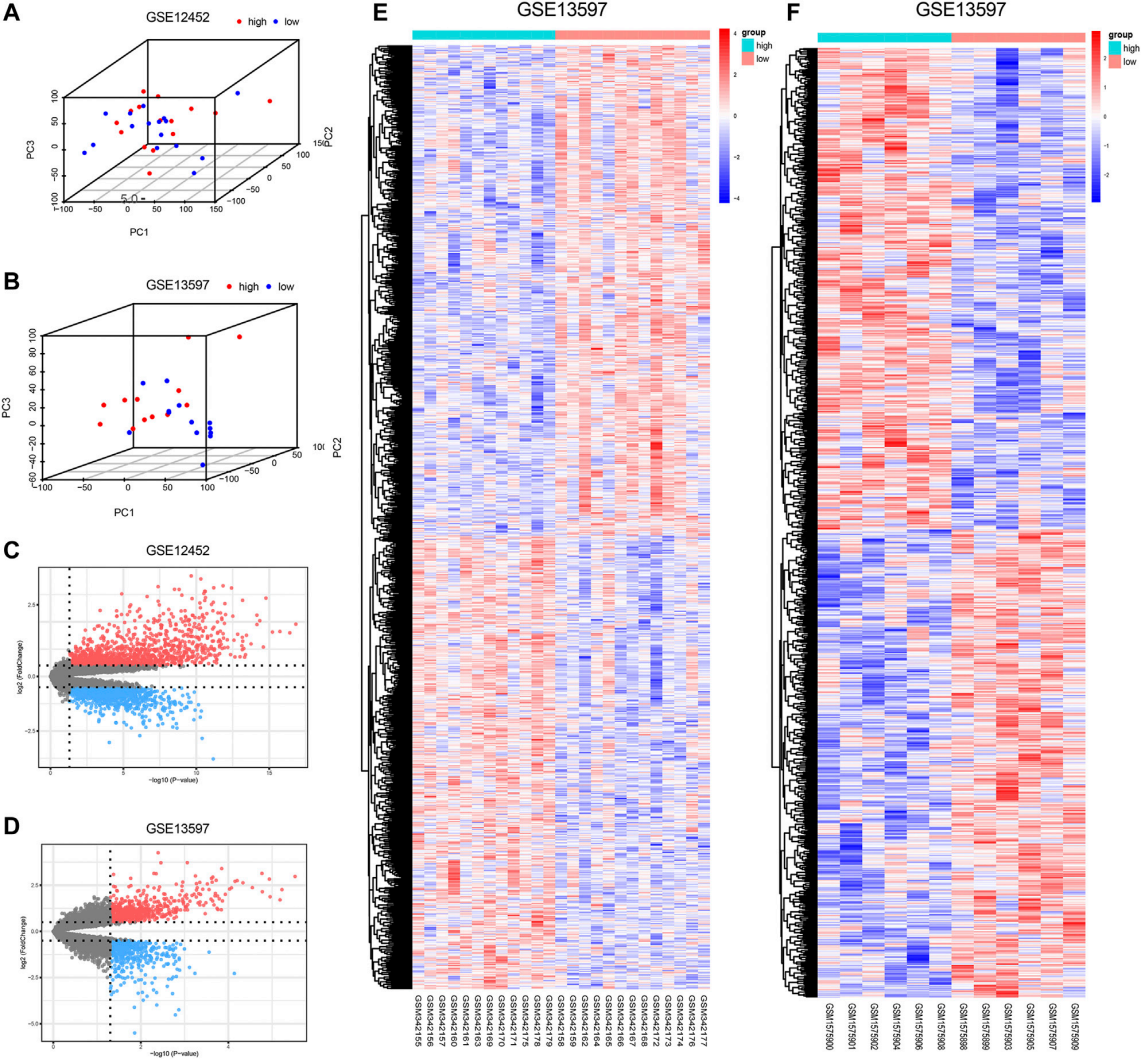
Differential expression after grouping by *AURKA.* **(A–B)**. 3DPCA cluster diagram of *AURKA* high and low expression groups; **(C–D)**. Volcano plots of DEGs; red represents up-regulated DEGs and blue represents down-regulated DEGs. Grey indicates genes with no differential expression; **(E–F)**. Heat map of the DEGs; red represents up-regulation, and blue represents down-regulation. *AURKA*, Aurora kinase A; PCA, principal component analysis; DEGs, differentially expressed genes.

Tumor samples were divided into high- and low-expression groups based on the median expression level of *AURKA*. The results of PCA indicated obvious clustering in the two groups (Figures 3A,B). Subsequently, based on the differential analysis of high- and low-*AURKA* expression groups, 1,095 DEGs were identified in the GSE12452 dataset and 1,130 DEGs were identified in the GSE13597 dataset (Figures 3C–F).

### WGCNA analysis and functional analysis

**FIGURE 4:**
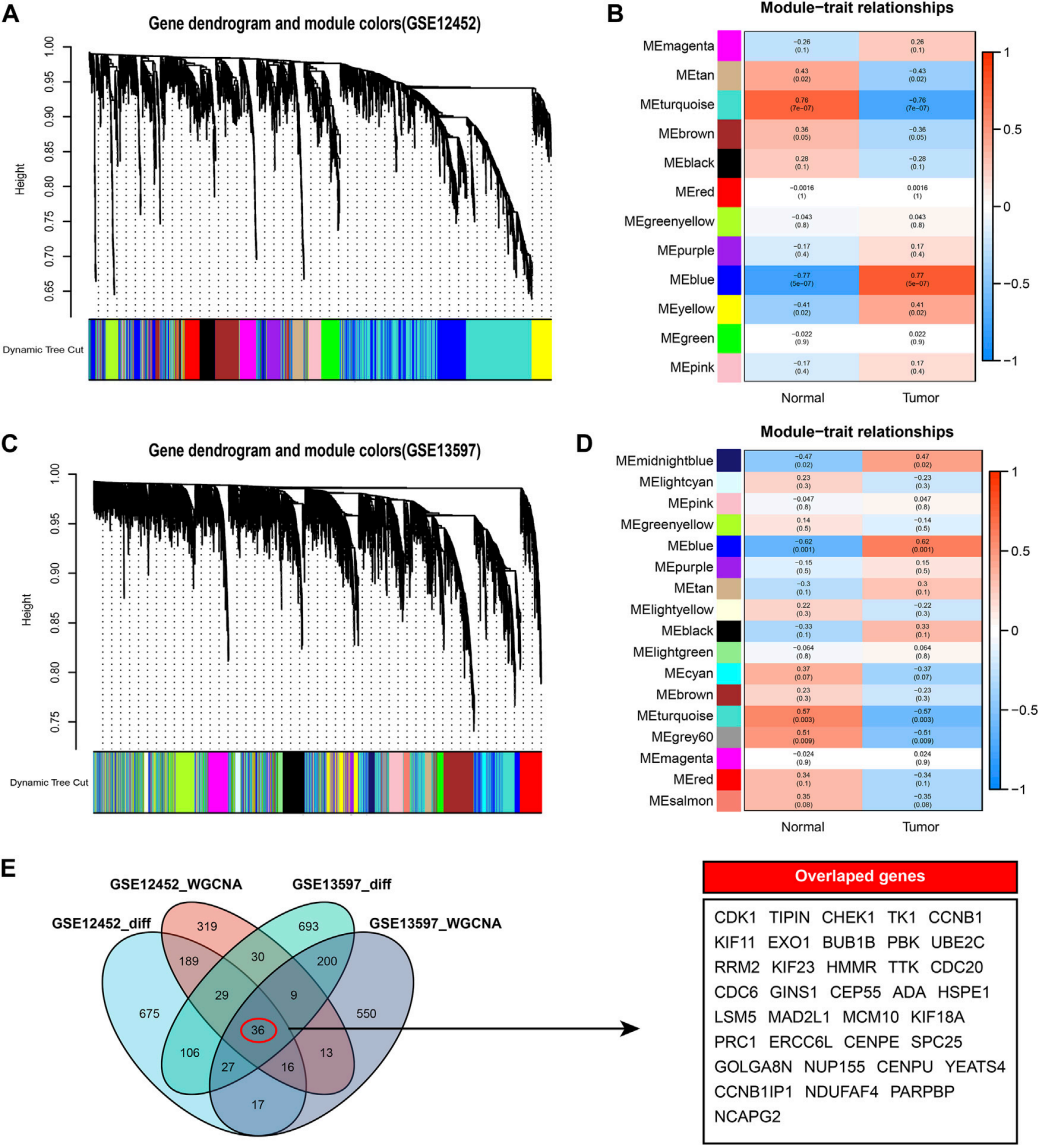
WGCNA co-expression network analysis. **(A–B)**. WGCNA analysis of tumor samples in the GSE12452 data set. A total of 12 significantly correlated modules were obtained, among which MEblue showed the strongest correlation with high expression of *AURKA*; **(C–D)**. WGCNA analysis of tumor samples in the GSE13597 data set. A total of 17 significantly correlated modules were obtained, among which MEblue showed the strongest correlation with high expression of *AURKA*; **(E)**. Venn diagram results of genes and differential genes of the most significant related modules in WGCNA. A total of 36 hub genes were identified. WGCNA, Weighted Gene Co-Expression Network Analysis; TOM, Topological overlap matrix; ME, Module eigengene; *AURKA*, Aurora kinase A.

To further identify the hub genes in the high and low *AURKA* groups, we performed WGCNA co-expression network analysis on GSE12452 and GSE13598 tumor samples. By associating module eigengenes with grouping information, we identified 12 feature modules in GSE12452, of which 4 were significantly positively correlated, and 8 were significantly negatively correlated (Figures 4A– B). Seventeen feature modules were identified in GSE13597, including 9 significant positive correlation modules and 8 significant negative correlation modules (Figures 4C– D). The larger the correlation coefficient of the module, the greater its correlation with *AURKA* expression. Then, we selected the most relevant MEblue modules in GSE12452 and GSE13597, respectively, and intersected the modules’ genes with the previous DEGs. Finally, 36 hub genes were obtained (Figures 4E–F).

**FIGURE 5:**
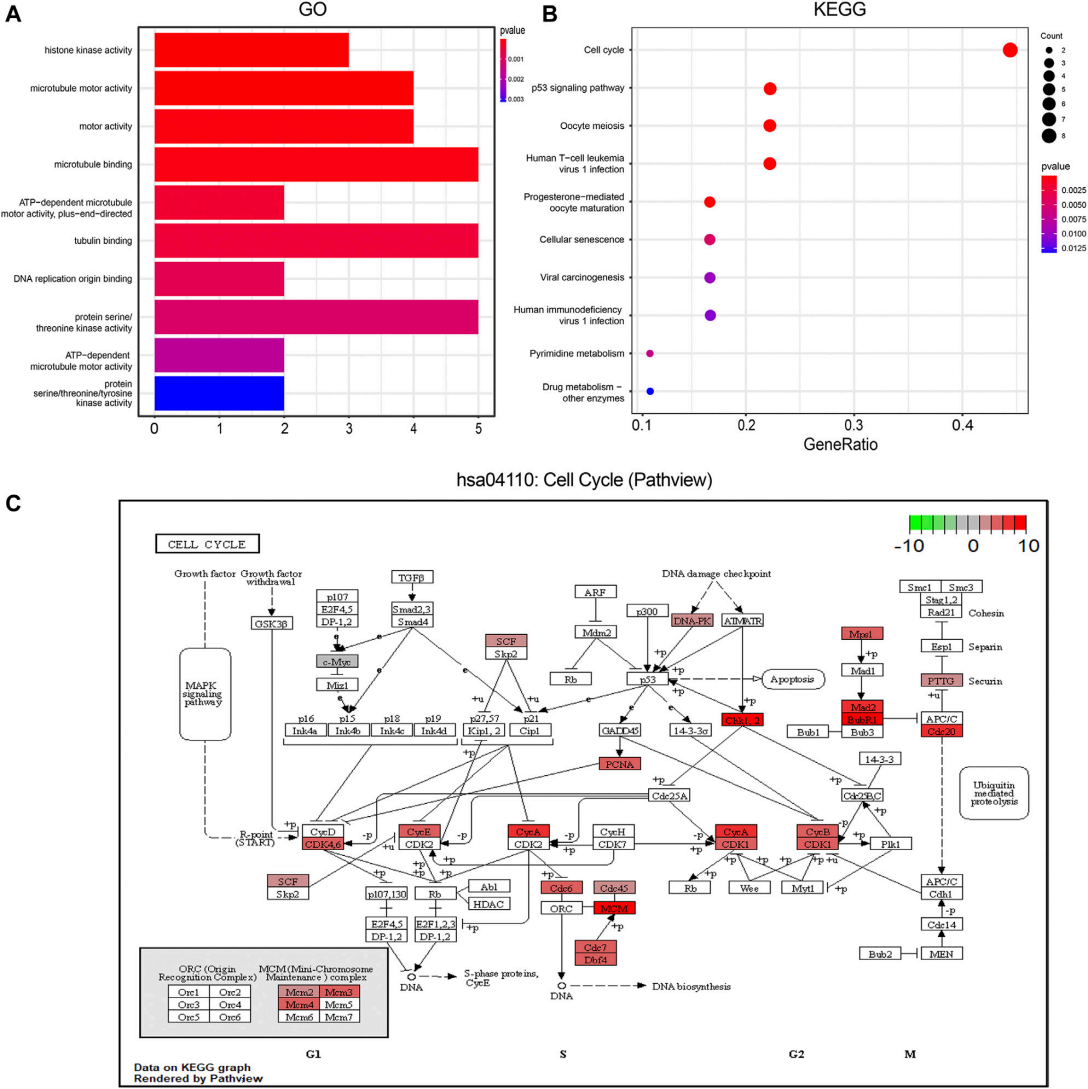
Function enrichment analysis of Hub genes. **(A)**. GO enrichment analysis. The color of the columns indicates the corrected *p* value; longer column indicates higher number of enriched genes; **(B)**. KEGG enrichment analysis; the annotation is the same as before; **(C)**. The most significantly enriched pathway in the KEGG pathway has04110: cell cycle pathway map display; red represents the degree of enrichment of genes, and the darker the shade, the more up-regulated the gene is in the pathway; the darker the shade of green color, the more obvious is the down-regulation of the gene. GO, Gene ontology; KEGG, Kyoto Encyclopedia of Genes and Genomes.

GO analysis revealed a close relation of these 36 hub genes with histone kinase activity, microtubule motor activity, and motor activity (Figure 5A). KEGG analysis showed that the pathways enriched by hub genes mainly included the cell cycle, p53 signaling pathway, Oocyte meiosis, and Human T-cell leukemia virus 1 infection (Figure 5B). Using the Pathview package, we identified hsa04110: cell cycle pathway as having the most significant enrichment. The results of GO and KEGG enrichment analysis related to the hub genes are shown in Supplementary Tables 3–4.

**FIGURE 6:**
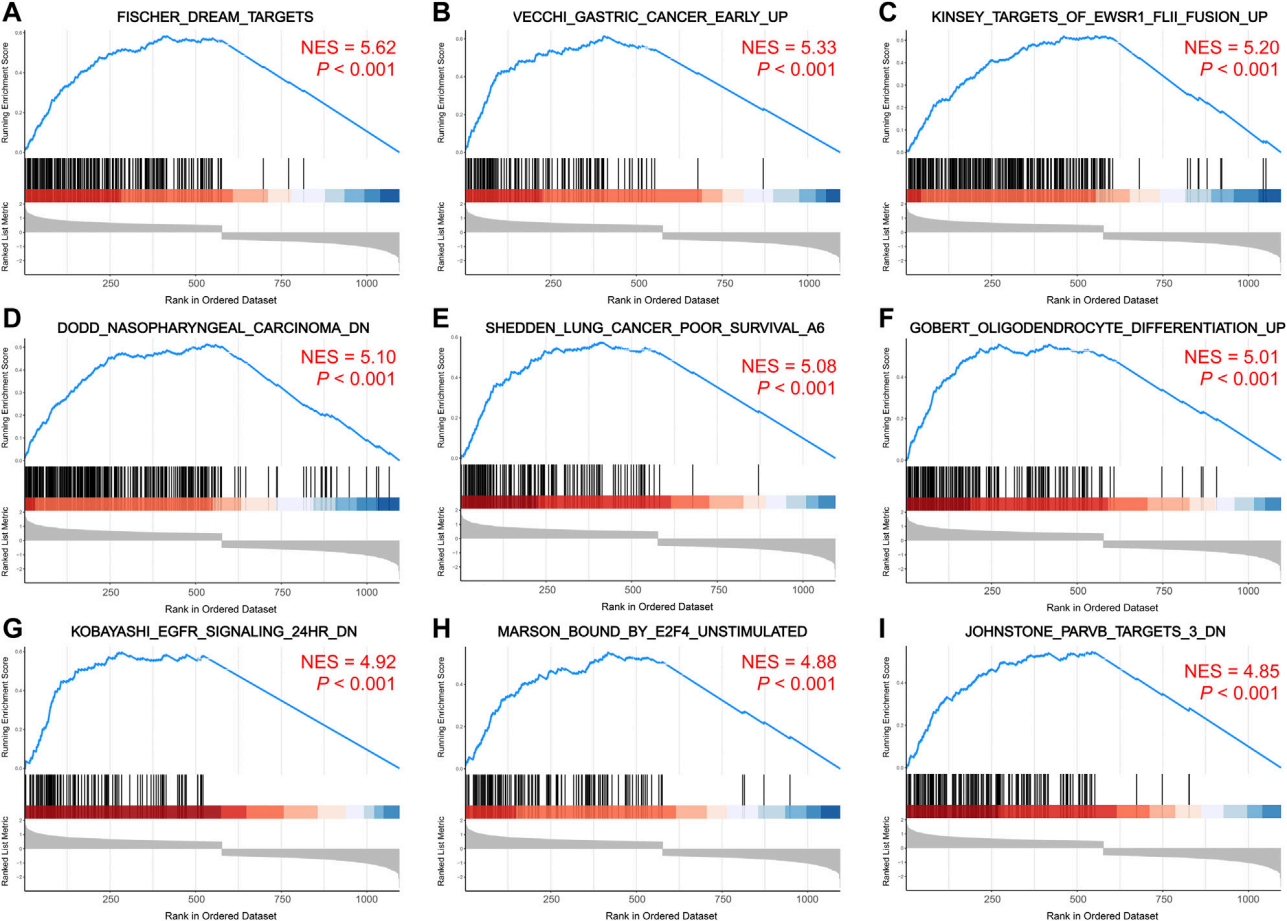
GSEA enrichment analysis of Hub genes. **(A–I)**. Top 9 pathways enriched for hub genes. The larger the NES value, the more genes are enriched in the pathway. GSEA, gene set enrichment analysis; NES, normalized enrichment score.

**FIGURE 7:**
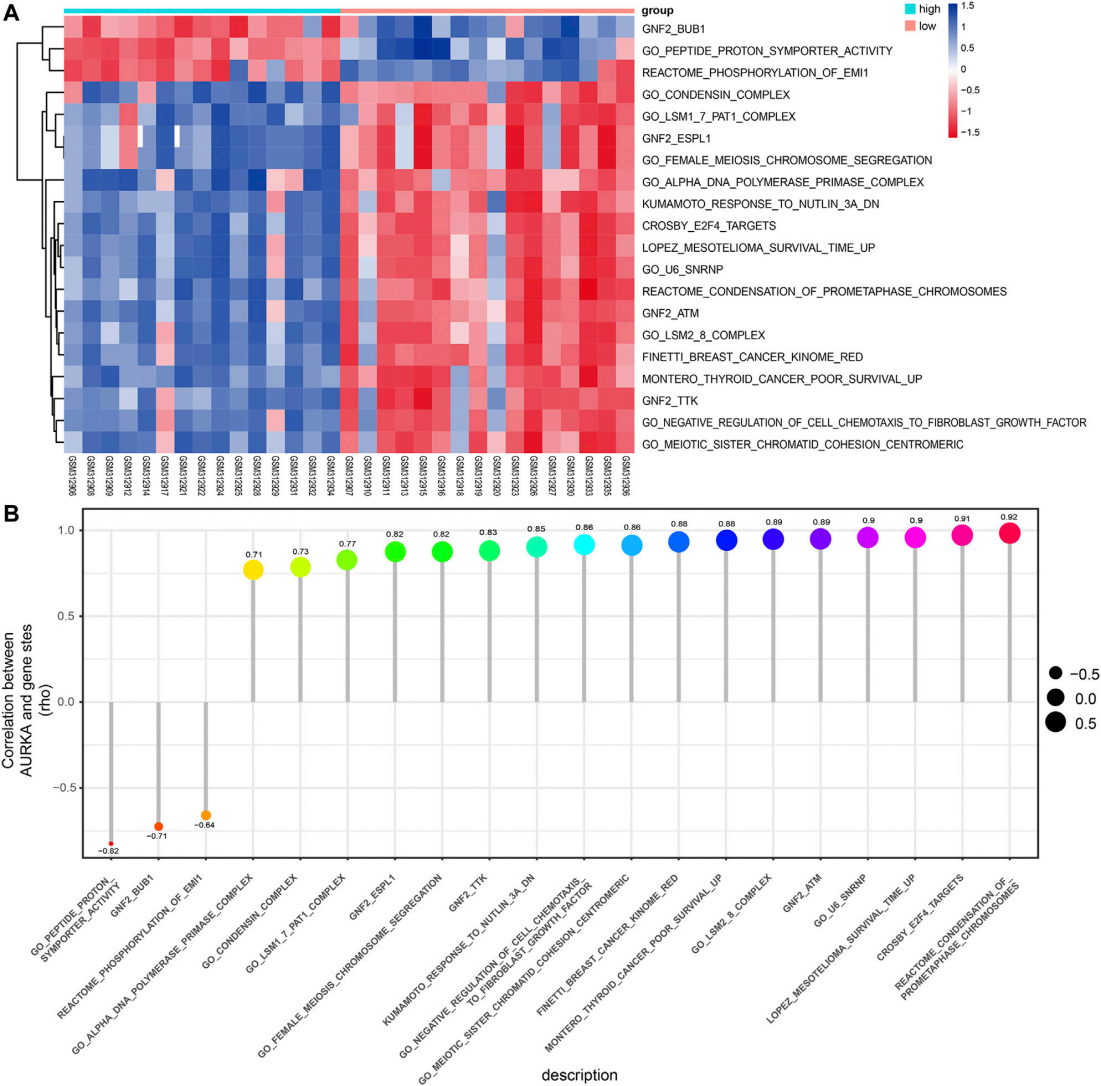
GSVA analysis of the Hub genes. **(A)**. Heatmap showing differential pathway heatmap quantified by GSVA. Red represents high enrichment, and blue indicates the opposite; **(B)**. Correlation of *AURKA* gene with differential pathways. Larger points are more relevant. GSVA, gene set variation analysis; *AURKA*, Aurora kinase **(A)**.

The pathways enriched by GSEA mainly involved FISCHER_DREAM_TARGETS, DODD_NASOPHARYNGEAL_CARCINOMA_DN, KINSEY_TARGETS_OF_EWSR1_FLII_FUSION_UP, SHEDDEN_LUNG_CANCER_POOR_SURVIVAL_A6 (Figures 6A–I). The GSVA enrichment results mainly included KUMAMOTO_RESPONSE_TO_NUTLIN_3A_DN, FINETTI_BREAST_CANCER_KINOME_RED, GO_NEGATIVE_REGULATION_OF_CELL_CHEMOTAXIS_TO_FIBROBLAST_GROWTH_FACTOR, etc. (Figure 7A). On further analysis of the expression and enrichment pathways of *AURKA*, we found that *AURKA* had the highest correlation with REACTOME_CONDENSATION_OF_PROMETAPHASE_CHROMOSOMES (Figure 7B). The results of GSEA of the top 20 NES of Hub genes are shown in Supplementary Table S5.

### PPI network and miRNA-target gene regulatory network

**FIGURE 8:**
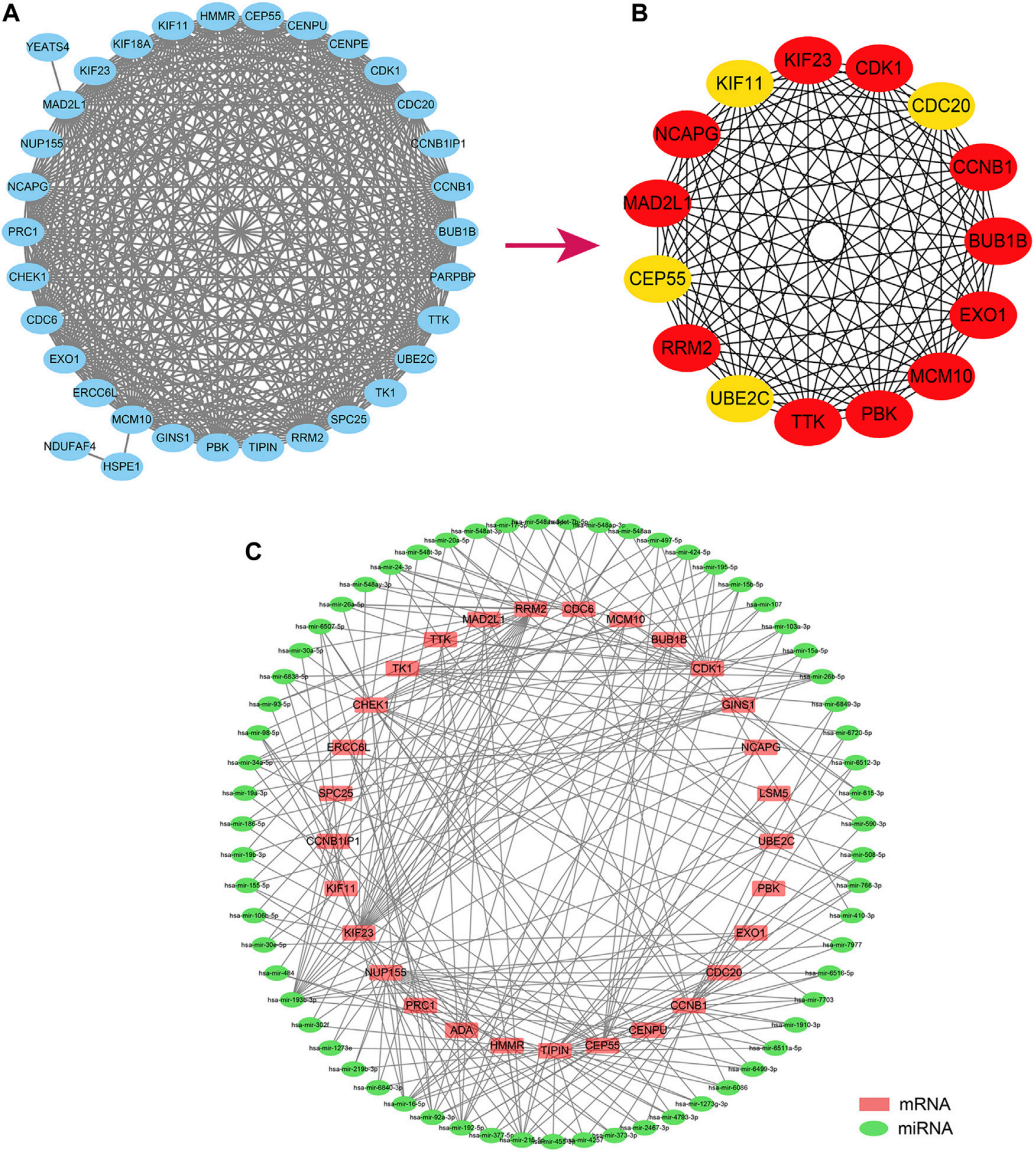
Analysis of the Hub gene regulation. **(A)**. The hub gene establishes the PPI regulatory network. **(B)**. The Cytohubba plugin further screened fifteen core genes; **(C)**. The miRNA-hub gene regulatory network regulates the hub gene predicted according to the Networkanalyst database. PPI, Protein-Protein Interaction Networks.

We constructed a PPI network from the String database to unravel the potential links between the 36 hub genes. On setting the minimum interaction score at 0.4, only 33 of the 36 hub genes were found to interact with the other gene pairs (Figure 8A). The PPI network consisted of these 33 eigengenes and 358 edges, with an average node degree of 19.9. We then further identified the most relevant genes in the PPI network using the Cytohubba plugin. Cytohubba detected the following 15 genes that could be considered as hubs: *KIF23, CDK1, CDC20, CCNB1, BUB1B, EX O 1, MCM10, PBK, TTK, UBE2C, RRM2, CEP55, MAD2L1, NCAPG,* and *KIF11* (Figure 8B).

Subsequently, we predicted the potential miRNAs regulating the 36 hub genes through the Networkanalyst database. The screening condition was “Trim current network to a minimum connected network.” The final sub-network contained 33 nodes, 171 edges, and 33 seeds (Figure 8C).

### Immune infiltration and correlation analysis

**FIGURE 9:**
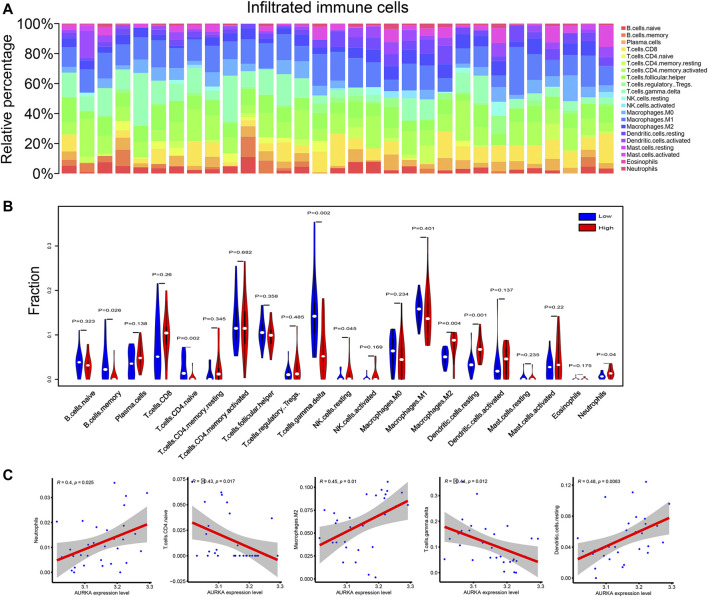
Immune infiltration correlation analysis. **(A)**. Panorama of immune cell infiltration in 21 tumor samples; **(B)**. Violin plot comparing immune cell infiltration between *AURKA* high and low expression groups; **(C)**. Correlation diagram of *AURKA* gene expression and immune cell infiltration. *AURKA*, Aurora kinase **(A)**.

To investigate the link between *AURKA* and immune cell infiltration, we calculated the proportion of these 21 cells infiltrating the tumor microenvironment using the CIBERSORT algorithm. Figure 9A exhibits a panoramic view of immune cell infiltration in the tumor microenvironment of NPC. As seen in the violin plot (Figure 9B), the infiltration of macrophages M2, neutrophils, and dendritic cells resting were relatively more in the *AURKA* high-expression group. In contrast, T-cell CD4^+^ naïve and T-cell γδ infiltration were less. Correlation analysis showed a positive correlation of *AURKA* expression with neutrophils, macrophages M2, and dendritic cells resting and negative correlation with T cell CD4^+^ naïve and T cell γδ cells (Figure 9C).

### Sensitivity analysis of drugs

**FIGURE 10:**
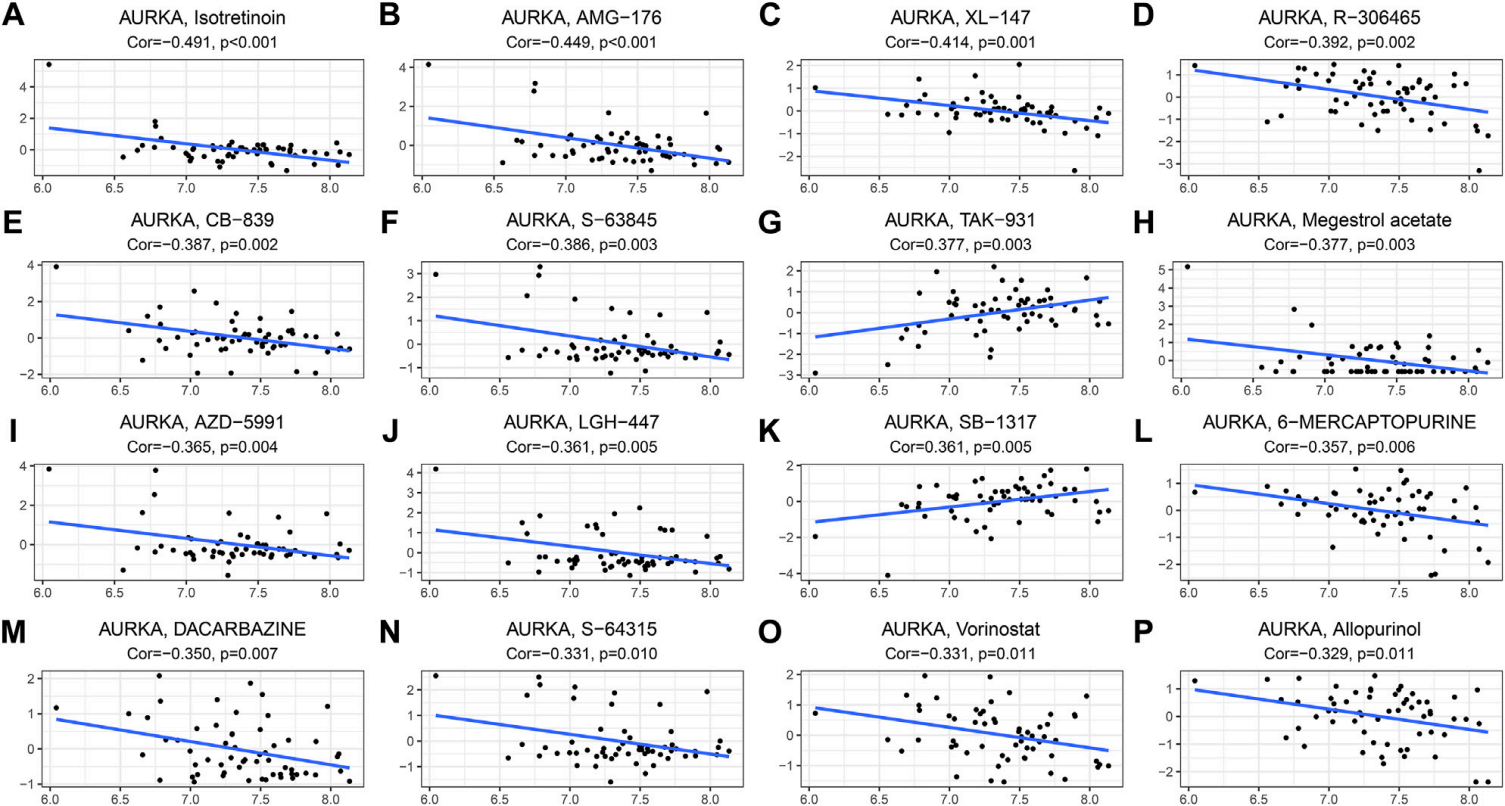
Drug sensitivity analysis. **(A–P)**. The top 16 drugs (or small molecule compounds) correlate with the most significant *AURKA* gene expression. *AURKA*, Aurora kinase A; NCI, National Cancer Institute.

The relationship between the *AURKA* gene and drug response was assessed by Pearson correlation analysis. In order of relevance, we selected the top 16 drugs that showed the strongest association with *AURKA*. We found negative correlation of *AURKA* with isotretinoin, AMG-176, XL-147, R-306465, CB-839, S-63845, megestrol acetate, AZD-5991, LGH-447, 6-Mercaptopurine, dacarbazine, S-64315, vorinostat, and allopurinol, and a positive correlation of AURKA with TAK-931 and SB-1317 (Figure 10A-P). Supplementary Table S6 shows the correlation between *AURKA* and drugs based on CellMiner.

### Tissue verification results of *AURKA*

**FIGURE 11:**
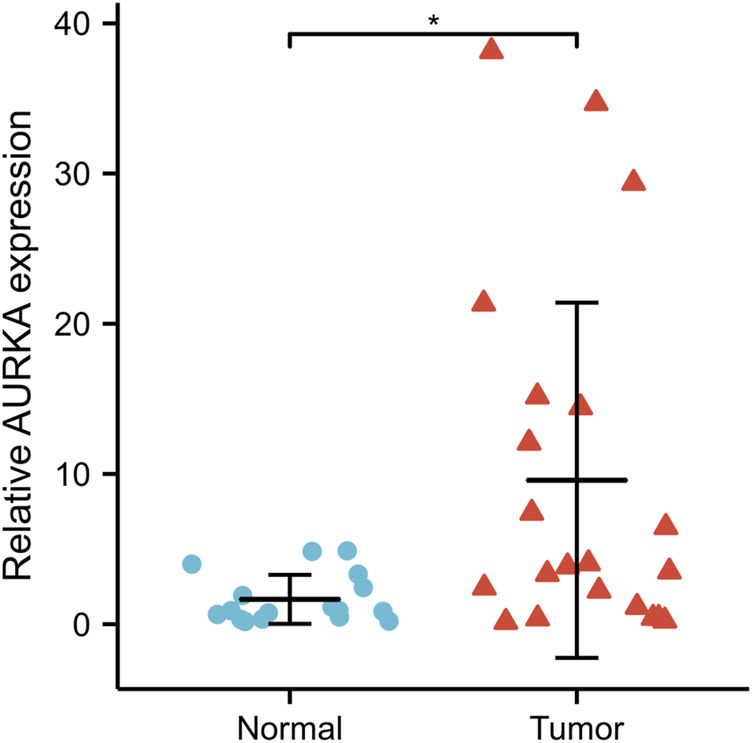
qRT-PCR validated *AURKA* expression in NPC tissue (*n* = 21) and normal nasopharyngeal epithelial tissue (*n* = 17). *p < 0.05. *AURKA*, Aurora kinase A; NPC, nasopharyngeal carcinoma.

Through qRT-PCR, the expression of *AURKA* was found to be significantly higher in cancer tissues compared to normal tissues (Wilcoxon rank-sum test, *p* < 0.05) (Figure 11).

## Discussion

Globally, an estimated 380,000–550,000 people die from head and neck cancer (HNC) every year (Khetan et al., 2019). NPC is one of the most common HNCs, accounting for 23.8% of all cases (Bray et al., 2018). Local radiation, surgery, other combination therapies, and concurrent chemotherapy can help achieve good disease control. However, 20%–30% patients develop local recurrence or distant metastasis (Pan et al., 2016), and these patients have a poor prognosis (median survival time: approximately 20 months) (Wei and Sham, 2005). Therefore, exploration of the specific mechanisms and key genes involved in NPC recurrence and metastasis is a key imperative to provide new ideas for identifying novel therapeutic targets.

Last few years have witnessed rapid progress in the research on NPC. However, no definite therapeutic targets and mechanism have been identified for treatment of this disease. *AURKA* has been shown to be highly expressed in certain malignant tumors and is potentially involved in the prophase of mitosis to promote G2/M transition (Katsha et al., 2015). Furthermore, *AURKA* has been shown to promote tumor progression and drug resistance by activating oncogenic signaling pathways and inhibiting the key tumor suppressor functions of p53 and TAp73 (Katsha et al., 2017; Wang et al., 2017; Wang-Bishop et al., 2019). However, the role of *AURKA* in NPC is not well characterized. We conducted comprehensive analysis of *AURKA* to illustrate its clinical significance and potential role in NPC.

We explored the potential role of *AURKA* in NPC evolution by analyzing tumor tissues and normal tissues in the GSE12452 and GSE13597 datasets. The *AURKA* gene was found to be differentially expressed in both datasets. Therefore, we chose *AURKA* as the research object and analyzed its potential functions and mechanisms using various bioinformatics methods. Our analysis revealed significant overexpression of *AURKA* in NPC tissues. The results of ROC curve analysis indicated that the high- and low-*AURKA* expression groups were highly discriminative. This indicates that *AURKA* plays an important role in the evolution of NPC.

Interestingly, similar results have been obtained in other studies. Upregulation of *AURKA* has been reported in various cancers, including neuroblastoma, lymphoma, colorectal, ovarian and prostate cancers (Lens et al., 2010; Willems et al., 2018). *AURKA* has been found to interact with various substrate proteins during DNA replication, such as the tumor suppressor gene p53, heterochromatin protein 1γ (HP1γ), histone H3, and HDAC6. Functional diversity makes *AURKA* an important drug target (Mou et al., 2021). We performed a drug sensitivity analysis on AURKA. In order to rank our top 16 antitumor drugs with the strongest associations, 14 of them were inversely associated with AURKA. Through analysis, it was found that AURKA may be an important drug target of dacarbazine, R-306465 and vorinostatz. KEGG analysis showed that the pathway with the most enriched genes was the cell cycle pathway. We speculate that interfering with the cell cycle of tumor cells by drugs can inhibit the development of tumors. Among them, dacarbazine mainly acts on inhibit the synthesis of purine, RNA and protein in the G2 phase, and it also affects the synthesis of DNA, which can inhibit the division of tumor cells. Both R-306465 and vorinostat were histone deacetylase (HDAC) inhibitors that exert tumor suppressive effects by inducing cell differentiation, cell regulation, and blocking the cell cycle. Therefore, we speculate that these three AURKA-related drugs can be applied to the treatment of NPC.

To further identify the hub genes in the high- and low-*AURKA* groups, we performed WGCNA co-expression network analysis on GSE12452 and GSE13598 tumor samples, respectively. Ultimately, we focused on 36 hub genes. Functional enrichment analysis of these hub genes indicated their potential involvement in the deterioration of NPC via histone kinase activity, microtubule motility activity, and cell cycle, p53 signaling pathway. Our findings are consistent with previous reports which showed that *AURKA* promotes the G2/M transition by regulating centrosome microtubule elongation and interacting with histone H3 during DNA replication (Giet et al., 1999; Kinoshita et al., 2005; Katsha et al., 2015; Mou et al., 2021).

Moreover, *AURKA* has been shown to participate in cancer-promoting processes by regulating the cell cycle and activating oncogenic signaling pathways, including p53 (Saiprasad et al., 2014; Mar et al., 2015; Vilgelm et al., 2015; Katsha et al., 2017; Wang et al., 2017; Wang-Bishop et al., 2019; Wang et al., 2021). Our findings are similar to those described above. In addition, we performed GSEA and GSVA to study the enriched gene set and key pathways. The results showed that GSVA enriched pathways mainly included chromosome condensation in the prophase and metaphase of the cell cycle. The GSEA was mainly enriched in nasopharyngeal carcinoma-related pathways. We speculate that *AURKA* is a promising molecular target for NPC therapy.

Subsequently, we investigated the role of *AURKA* in NPC by immune infiltration correlation analysis and drug sensitivity analysis. Correlation analysis of immune infiltrating cells showed that tissues with high *AURKA* expression had greater infiltration of neutrophils, macrophages M2, and dendritic cells resting, but less infiltration of T cell CD4^+^ naïve and T cell γδ. The tumor microenvironment can induce the polarization of macrophages towards the M2 phenotype (Mantovani et al., 2002; Ma et al., 2010). M2 macrophages directly inhibit CD4^+^ T cell-mediated tumor killing through cell-cell contact or release TGF-β and IL-10 and accelerate lymphatic tumor metastasis (Kurahara et al., 2011). High infiltration of M2 macrophages predicts poor tumor prognosis. T cell γδ cells are immune cells that kill cancer cells and tumor stem cells and recognize cancer antigens. Less infiltration of T cell γδ in tumor tissue predicts poor prognosis. Taken together, we found that high AURKA expression may predict poor prognosis in NPC.

Some limitations of our study should be considered while interpreting the results. First, further experiments are required to verify the biological mechanism of *AURKA*. Second, this study lacked corresponding clinical correlation studies, which are essential to determine the potential role of AURKA as a novel therapeutic target. In addition, the batch-to-batch variation that cannot be avoided and removed may be created during data analysis. Finally, this study has a relatively small sample size and lacks tissue samples with early stage in NPC, and tissue samples should be increased to further analyze the correlation of *AURKA* expression with clinical characteristics and prognosis.

## Conclusion

This study comprehensively explored the potential molecular mechanisms and functional roles of *AURKA* in the evolution of NPC through a variety of databases and bioinformatics analysis methods. We verified that *AURKA* is highly expressed in NPC tissues. Most importantly, the current analysis supports an oncogenic role of *AURKA* in the context of NPC, which may be a potential therapeutic target. Our research points out a new direction for molecular targeted therapy for NPC. Nevertheless, the specific mechanism and molecular targets of *AURKA* in NPC still need further experimental verification.

## Data Availability

The original contributions presented in the study are included in the article/Supplementary Materials, further inquiries can be directed to the corresponding authors.
