# Supplementary material for: Predicting AURKA as a novel therapeutic target for NPC: A comprehensive analysis based on bioinformatics and validation
Source: Front Genet. 2022 Aug 22;13:926546. doi: 10.3389/fgene.2022.926546 (PMC9441489; doi:10.3389/fgene.2022.926546)
Supplement: Supplementary file 1 [file DataSheet1.docx]

**Supplemental Table 1. Clinical data for qRT-PCR of patients with nasopharyngeal carcinoma in our institution**

| Characteristic | levels | Overall |
| --- | --- | --- |
| N |  |  |
| Gender, n (%) | Female | 3(14.3%) |
|  | Male | 18(85.7%) |
| Age, n (%) | <40 | 4(19.0%) |
|  | >=40 | 17(81.0%) |
| Smoking, n (%) | Yes | 8(38.1%) |
|  | No | 13(61.9%) |
| EBV-DNA, n (%) | <500 | 3(14.3%) |
|  | >500 | 18(85.7%) |
| Tumor stage, n (%) | 1 | 1(4.8%) |
|  | 2 | 2(9.5%) |
|  | 3 | 6(28.6%) |
|  | 4 | 12(57.1%) |
| Node stage, n (%) | 0 | 2(9.5%) |
|  | 1 | 6(28.6%) |
|  | 2 | 5(23.8%) |
|  | 3 | 8(38.1%) |
| Pathologic TNM stage, n (%) | III | 3(14.3%) |
|  | IVa | 16(76.2%) |
|  | IVb | 2(9.5%) |

**Supplemental Table 2. The primers of *AURKA* and 18S-rRNA**

| **Primers** | **Forward primers（5’to 3’）** | **Reverse primers（5’to 3’）** |
| --- | --- | --- |
| qPCR-AURKA | CTCATCCTGTCTCCAGGCCA | TTACCCAGAGGGCGACCAAT |
| qPCR-18S-rRNA | GCAATTATTCCCCATGAACG | GGGACTTAATCAACGCAAGC |

| **Supplemental Table 3. Significant enriched GO terms and pathways of hub genes** | | | | |
| --- | --- | --- | --- | --- |
| **ID** | **Description** | **P.adjust** | **geneID** |  |
| GO:0035173 | histone kinase activity | 0.0003338 | CDK1/CHEK1/CCNB1 |  |
| GO:0003777 | microtubule motor activity | 0.0005191 | KIF11/CENPE/KIF18A/KIF23 |  |
| GO:0003774 | motor activity | 0.0025673 | KIF11/CENPE/KIF18A/KIF23 |  |
| GO:0008017 | microtubule binding | 0.0025673 | KIF11/CENPE/KIF18A/KIF23/PRC1 |  |
| GO:0008574 | ATP-dependent microtubule motor activity, plus-end-directed | 0.0067654 | KIF11/KIF18A |  |
| GO:0015631 | tubulin binding | 0.0075126 | KIF11/CENPE/KIF18A/KIF23/PRC1 |  |
| GO:0003688 | DNA replication origin binding | 0.0101058 | CDC6/MCM10 |  |
| GO:0004674 | protein serine/threonine kinase activity | 0.012405 | CDK1/CHEK1/TTK/PBK/BUB1B |  |
| GO:1990939 | ATP-dependent microtubule motor activity | 0.0172079 | KIF11/KIF18A |  |
| GO:0004712 | protein serine/threonine/tyrosine kinase activity | 0.0269841 | TTK/PBK |  |
| GO:0008022 | protein C-terminus binding | 0.0397714 | CDC20/YEATS4/MAD2L1 |  |

| **Supplemental Table 4. Results of KEGG enrichment analysis of the hub genes** | | | | |  |
| --- | --- | --- | --- | --- | --- |
| **ID** | **Description** | **P.adjust** | | **geneID** | |
| hsa04110 | Cell cycle | 2.21E-09 | 983/1111/991/891/7272/990/701/4085 | |  |
| hsa04115 | p53 signaling pathway | 0.0002022 | 983/1111/6241/891 | |  |
| hsa04114 | Oocyte meiosis | 0.0012612 | 983/991/891/4085 | |  |
| hsa05166 | Human T-cell leukemia virus 1 infection | 0.0062395 | 1111/991/701/4085 | |  |
| hsa04914 | Progesterone-mediated oocyte maturation | 0.0062395 | 983/891/4085 | |  |
| hsa04218 | Cellular senescence | 0.0184619 | 983/1111/891 | |  |
| hsa00240 | Pyrimidine metabolism | 0.0229124 | 7083/6241 | |  |
| hsa05203 | Viral carcinogenesis | 0.0287242 | 983/1111/991 | |  |
| hsa05170 | Human immunodeficiency virus 1 infection | 0.0287242 | 983/1111/891 | |  |
| hsa00983 | Drug metabolism - other enzymes | 0.0318914 | 7083/6241 | |  |

| **Supplemental Table 5. GSEA enrichment analysis of hub genes (NES top 20)** | | | |  |
| --- | --- | --- | --- | --- |
| **ID** | **setSize** | **NES** | ***P*.adjust** | |
| FISCHER_DREAM_TARGETS | 195 | 5.6843599 | 4.91E-09 | |
| VECCHI_GASTRIC_CANCER_EARLY_UP | 125 | 5.4176259 | 4.91E-09 | |
| DODD_NASOPHARYNGEAL_CARCINOMA_DN | 265 | 5.1469241 | 4.91E-09 | |
| KINSEY_TARGETS_OF_EWSR1_FLII_FUSION_UP | 238 | 5.1355935 | 4.91E-09 | |
| SHEDDEN_LUNG_CANCER_POOR_SURVIVAL_A6 | 136 | 5.124537 | 4.91E-09 | |
| GOBERT_OLIGODENDROCYTE_DIFFERENTIATION_UP | 152 | 5.023951 | 4.91E-09 | |
| KOBAYASHI_EGFR_SIGNALING_24HR_DN | 105 | 4.952582 | 4.91E-09 | |
| JOHNSTONE_PARVB_TARGETS_3_DN | 142 | 4.9352372 | 4.91E-09 | |
| MARSON_BOUND_BY_E2F4_UNSTIMULATED | 132 | 4.9169473 | 4.91E-09 | |
| FLORIO_NEOCORTEX_BASAL_RADIAL_GLIA_DN | 89 | 4.8795886 | 4.91E-09 | |
| MODULE_54 | 110 | 4.8440956 | 4.91E-09 | |
| RODRIGUES_THYROID_CARCINOMA_POORLY_DIFFERENTIATED_UP | 133 | 4.8218638 | 4.91E-09 | |
| CAIRO_HEPATOBLASTOMA_CLASSES_UP | 127 | 4.7826658 | 4.91E-09 | |
| WEI_MYCN_TARGETS_WITH_E_BOX | 135 | 4.7266466 | 4.91E-09 | |
| GSE15750_DAY6_VS_DAY10_EFF_CD8_TCELL_UP | 80 | 4.7168356 | 4.91E-09 | |
| SOTIRIOU_BREAST_CANCER_GRADE_1_VS_3_UP | 83 | 4.6059925 | 4.91E-09 | |
| RODRIGUES_THYROID_CARCINOMA_ANAPLASTIC_UP | 111 | 4.5978192 | 4.91E-09 | |
| DUTERTRE_ESTRADIOL_RESPONSE_24HR_UP | 98 | 4.5389736 | 4.91E-09 | |
| BASAKI_YBX1_TARGETS_UP | 86 | 4.5295473 | 4.91E-09 | |
| ROSTY_CERVICAL_CANCER_PROLIFERATION_CLUSTER | 84 | 4.5153357 | 4.91E-09 | |

| **Supplemental Table 6. Correlation between *AURKA* and drugs based on CellMiner** | | | |
| --- | --- | --- | --- |
| **Gene** | **Drug** | **Correlation** | ***P* value** |
| AURKA | Isotretinoin | -0.490700074 | 7.96E-05 |
| AURKA | AMG-176 | -0.449053319 | 0.000360454 |
| AURKA | XL-147 | -0.414126714 | 0.001111609 |
| AURKA | R-306465 | -0.392175134 | 0.002126402 |
| AURKA | CB-839 | -0.387341462 | 0.002438732 |
| AURKA | S-63845 | -0.385752491 | 0.002549982 |
| AURKA | TAK-931 | 0.376891443 | 0.003257244 |
| AURKA | Megestrol acetate | -0.376523242 | 0.003290071 |
| AURKA | AZD-5991 | -0.36515343 | 0.004459355 |
| AURKA | LGH-447 | -0.360822339 | 0.004993074 |
| AURKA | SB-1317 | 0.360565827 | 0.005026377 |
| AURKA | 6-MERCAPTOPURINE | -0.356861447 | 0.005529581 |
| AURKA | DACARBAZINE | -0.350222538 | 0.006542863 |
| AURKA | S-64315 | -0.331309589 | 0.010370434 |
| AURKA | Vorinostat | -0.330534532 | 0.010561911 |
| AURKA | Allopurinol | -0.32880233 | 0.011000945 |
| AURKA | TPX-0005 | 0.327667131 | 0.011297151 |
| AURKA | BAY-1251152 | 0.323362767 | 0.012483758 |
| AURKA | E-7820 | -0.321370205 | 0.01306847 |
| AURKA | Fluphenazine | -0.319376298 | 0.013677022 |
